# Perinatal Programming of Asthma: The Role of Gut Microbiota

**DOI:** 10.1155/2012/932072

**Published:** 2011-11-03

**Authors:** Meghan B. Azad, Anita L. Kozyrskyj

**Affiliations:** Department of Pediatrics, University of Alberta, Edmonton Clinic Health Academy, 11405 87th Avenue, Edmonton, Alberta, Canada T6G IC9

## Abstract

Perinatal programming, a dominant theory for the origins of cardiovascular disease, proposes that environmental stimuli influence developmental pathways during critical periods of prenatal and postnatal development, inducing permanent changes in metabolism. In this paper, we present evidence for the perinatal programming of asthma via the intestinal microbiome. While epigenetic mechanisms continue to provide new explanations for the programming hypothesis of asthma development, it is increasingly apparent that the intestinal microbiota plays an independent and potentially interactive role. Commensal gut bacteria are essential to immune system development, and exposures disrupting the infant gut microbiota have been linked to asthma. This paper summarizes the recent findings that implicate caesarean delivery, breastfeeding, perinatal stress, probiotics, and antibiotics as modifiers of infant gut microbiota in the development of asthma.

## 1. Introduction

Supported by evidence from farm lifestyle and endotoxin studies [1], the hygiene hypothesis has changed our understanding of the environmental origins of asthma and allergic disease. However, it fails to explain the coexisting epidemic in autoimmune disease or the high rates of asthma among the urban poor in the United States. This limitation has motivated the continued search for alternate explanations such as the microflora hypothesis [2] and the developmental origins hypothesis for health and disease (DOHaD) [3]. Originating as the Barker hypothesis, DOHaD or “perinatal programming” has been a dominant theory for the association between low birth weight and cardiovascular or metabolic disease in later life [3]. In this paper, we present evidence for the perinatal programming of childhood asthma, with a focus on the intestinal microbiome. We begin with a discussion and examples of perinatal programming and epigenetics, highlighting environmental exposures during the *in utero* and *ex utero* time periods that are potential stimuli for the early programming of asthma. More detailed discussion is provided on the postnatal development of immunity and its interaction with the intestinal microbiome, with evidence for the long-term impact of 5 perinatal exposures: caesarean section delivery, breastfeeding, antibiotics, probiotics, and perinatal stress.

## 2. Perinatal Programming of Disease and Epigenetics

The DOHaD hypothesis proposes that nutrition and other environmental stimuli or insults can influence developmental pathways during critical periods of prenatal, and postnatal development, and subsequently induce permanent changes in metabolism and disease susceptibility [3, 4]. While coined by Barker as the “fetal origins” hypothesis [5], the realization that human development extends into the postnatal period led to a change in nomenclature to the “developmental origins” hypothesis. “Programming” is another common term for the DOHaD hypothesis. The DOHaD approach was initially focused on early-life nutrition as a pathway for obesity and related metabolic abnormalities but has since been expanded to include the psychobiological effects of fetal and infant exposure to stress [3]. In fact, overexposure of the fetus to maternal stress and glucocorticoids has been proposed as an alternative to fetal undernutrition, to account for the association between the prenatal environment and the development of cardiovascular, metabolic, and neuroendocrine phenotypes.

Based on evolutionary concepts, the DOHaD theory surmises that predictive adaptive responses of the fetus to *in utero* environmental cues promote a phenotype that is optimally suited for the postnatal environment [6]. If the prediction is correct, there will be a good match between the adopted phenotype and the postnatal environment. If the prediction is poor, there will be a mismatch between the phenotype produced and the environment experienced, resulting in negative health consequences. For example, constrained fetal or infant growth from malnutrition followed by enhanced nutrition during infancy or later childhood leads to metabolic abnormalities, such as insulin resistance. Other DOHaD-informed studies have detected smaller brain hippocampal volume (a risk factor for depression and psychopathology) in individuals who were born low birth weight and exposed to postnatal adversity [3]. 

Epigenetic mechanisms—the imprinting of environmental experiences on infant gene expression—are increasingly thought to be at the root of the DOHaD hypothesis [7]. Specifically, epigenetic modifications affect gene expression without altering DNA sequence. There is strong evidence that early environmental exposures can activate or silence genes by altering DNA methylation, histone acetylation and methylation, and chromatin structure [3]. Since these modifications regulate the degree of DNA coiling and accessibility for transcription, they determine gene expression. DNA methylation is the best-characterized epigenetic modification [4], occurring at cytosine-guanine dinucleotides (CpGs). Site-specific and regional changes in CpG methylation are often highly correlated with gene expression. Following DNA replication, the original pattern of CpG methylation is restored, ensuring the perpetuation of epigenetic information in replicating cells. 

The epigenetic phenomenon is clearly demonstrated by evidence of diet-induced DNA methylation during mouse fetal development and subsequent changes to coat color and body weight in the offspring of mothers who consume a high-soy diet during pregnancy [8]. DNA hypomethylation has consistently been documented in rat models of intrauterine growth retardation [9]. In humans, assisted-reproduction studies have shown that inappropriate epigenetic reprogramming can increase the risk of some developmental syndromes [4]. Altered DNA methylation has also been observed in response to maternal undernutrition during pregnancy and following child abuse [9].

## 3. Perinatal Programming of Asthma

Since immune and lung development occur largely *in utero* and during early childhood [10], perinatal programming is a plausible pathway for allergic and respiratory disease [11, 12]. Indeed, fetal exposure to maternal smoking during pregnancy, separately from postnatal exposure to second-hand smoke, can increase risk for asthma in offspring [13, 14]. As described in the review by Hylkema and Blacquiere [13], evidence is accumulating to show that tobacco smoke can modify fetal lung development and immune function. Other intrauterine exposures, such as maternal stress or adherence to a Mediterranean diet (high in folic acid and antioxidants), are also known to modify the risk of allergic disease in the offspring [15, 16]. 

Recent studies show that prenatal exposures can activate or silence immune-related genes through epigenetic mechanisms. Breton et al. found significantly lower global methylation of DNA in young schoolchildren with *in utero* exposure to maternal smoking, with hypermethylation at specific gene loci [17], and several examples of diet-induced modification of DNA methylation have been provided in the recent review by Attig et al. [9]. For example, maternal folic acid supplementation has been found to increase methylation of the insulin-like growth factor 2 gene in offspring, and animal studies show that folic acid can prevent hypomethylation resulting from maternal undernutrition. While there is evidence that immune system development (specifically T-cell differentiation) is under epigenetic regulation [18, 19], and epigenetic changes (such as DNA methylation) have been found in children with asthma [20, 21], it remains to be determined whether epigenetic modifications mediate the effects of maternal smoking, stress, and diet on child asthma. 

As noted earlier, the DOHaD paradigm is not limited to the *in utero* time period. This brings us to the main focus of our paper: the role of gut microbiota in the perinatal programming of asthma. Mounting evidence indicates that the continuous and predictable presence of commensal bacteria (microbiota) in the human intestine plays an important role in shaping the immune system during infancy [22, 23]. Indeed, studies have shown that commensal gut microbes interact with immune cells to create and maintain host tolerance, influencing both innate and adaptive immune responses [24]. As detailed in later sections of the current paper, this “microflora hypothesis” has been put forward as an example of early-life programming of allergy and asthma [24, 25]. A key characteristic of metabolic programming or imprinting is the need to distinguish primary “imprints” from secondary physiological alterations that arise in response to primary imprints. This requires evidence that primary imprints are present directly after the programming period as well as in later life [4]. With this criterion in mind, we attempt to advance the DOHaD thesis of asthma by presenting evidence on how early-life environmental modifications of the intestinal microbiome can result in permanent changes to microbiota composition and immunity. 

## 4. Immune System Development and Gut Microbiota

Development of the immune system begins *in utero *and continues postnatally. Human lymphocytes first appear in the liver within several weeks of conception and are evident in the thymus by 10–12 weeks of gestation [26]. They are responsive to mitogen stimulation by the second trimester [27], and allergen-specific responses have been documented as early as 22 weeks gestation [28]. At birth, cytokine responses are dominated by T-helper cell type 2 (Th2) cytokines [29], and many aspects of neonatal immune function remain immature, including Th1 cytokine production, T-cell signaling and effector functions, monocyte responsiveness, and antigen presentation by dendritic cells [30]. 

Pregnancy itself is associated with a transient depression of maternal cell-mediated immunity [31] and predominance of Th2 cytokines at the maternofetal interface [32], which are thought to protect the fetus from immunologic rejection by the mother [33]. The maternal environment during pregnancy promotes Th2 polarity in the fetal immune system, with transition to a nonallergic Th1 phenotype occurring after birth. If this transition is delayed or impaired during early postnatal life, there is an increased risk of atopic disease including asthma [30]. 

Following birth, maternal influence on the developing infant immune system continues through breastfeeding. Maternal antibodies (including IgG and IgA) are transferred in breast milk, providing passive immunity to offspring during infancy. Immune cells (neutrophils and macrophages) and cytokines (interleukins, TNF*α*, and TGF*β*) are also present in breast milk, along with bactericidal enzymes and antiviral factors [34]. Nutrients and growth factors in breast milk have been shown to regulate the innate immunity [35], while fatty acid composition can modulate neonatal cytokine responses [36]. Despite the many protective factors transmitted in breast milk, it remains controversial whether breastfeeding is protective against asthma development in the infant. While several studies have shown that asthma risk is reduced in breast-fed infants [37–40], others claim there is no association [41, 42], and an inverse relationship has been demonstrated for children with maternal history of asthma [43, 44]. Wright et al. found that in school age children born to asthmatic mothers, longer duration of exclusive breastfeeding was associated with an increased risk of asthma [43]. These findings are supported by animal studies showing that breast milk can mediate the transmission of asthma risk from mother to offspring [45], possibly through the delivery of high concentrations of Th2 cytokines. Thus, while breastfeeding unquestionably provides nutritional and immunological benefits to the developing infant, its role in the perinatal programming of asthma remains controversial.

In addition to maternal immunogenic factors transferred during and after pregnancy, it is increasingly apparent that postnatal microbial exposure provides an essential source of immune stimulation. Colonization of the intestine begins during the birthing process, and mounting evidence indicates that these commensal bacteria play a central role in programming the neonatal immune system [23, 24]. For example, gut microbes have been shown to induce regulatory T cells that help guide the host's Th1/Th2 balance, and recognition of microbiota-derived peptides by mucosal receptors has been shown to enhance systemic innate immunity [24]. It has also been hypothesized that commensal gut microbes may produce metabolites capable of epigenetic modifications [46]; however, this remains to be proven experimentally. Microbial metabolites include short chain fatty acids [47], which could influence asthma development, since maternal and infant dietary fatty acid composition have been associated, albeit inconsistently, with childhood asthma [48]. 

Since the early 2000s, we have known that infants who ultimately develop allergic disease harbor a distinct gut microbiota [49, 50], and new evidence suggests this may also be true for asthma. Two birth cohort studies have reported that gut microbiota profiles in the first month of life can predict recurrent wheeze or possible asthma later in childhood [51, 52]. In particular, colonization with the pathogen *Clostridium difficile* has been associated with increased future risk of wheeze or asthma [51, 53]. Results from ongoing studies employing new “next generation” technologies are highly anticipated and promise to vastly improve our understanding of infant gut microbiota composition, including how it may contribute to asthma development. In parallel, researchers are increasingly focusing on exposures that influence the developmental programming of the intestinal microbiome.

## 5. Perinatal Programming of the Intestinal Microbiome

A groundbreaking international study has shown that adult gut microbiota can be classified according to a limited number of distinct microbial compositions or “enterotypes” that respond differently to diet and drug intake [54]. Enterotypes are likely established during early life, explaining why neonatal gut microbiota composition has a lasting effect on health and immunity [22]. Indeed, research has shown that gut microbiota profiles during infancy can predict overweight at school age [49], and accumulating evidence indicates that asthma prediction may also be possible [51, 52]. From the DOHaD perspective, these associations could reflect developmental “mismatch” scenarios, whereby disturbances to early-life gut microbiota cause the infant to be maladapted for future microbial exposures, leading to inappropriate immune responses that ultimately contribute to chronic disorders such as overweight or asthma. Consequently, there is growing interest in learning which environmental exposures influence microbiota development in the infant gut. The KOALA birth cohort studies in The Netherlands have identified several perinatal exposures that alter the intestinal microbiota at one month of age [55, 56]. In this paper, we have chosen to focus on 5 perinatal exposures, for which there is the most evidence regarding associations with the development of asthma (Table 1): caesarean section delivery, exclusivity of breastfeeding, use of antibiotics, use of probiotics, and perinatal stress.

### 5.1. Caesarean Section Delivery

The newborn's first microbial exposure is to maternal microbiota during birth, which lays the foundation for intestinal colonization. Caesarean section delivery prevents exposure to maternal fecal microbes, resulting in fewer intestinal Bifidobacteria and Bacteroides [57, 58]. In the absence of these commensal species, infants delivered by caesarean section are more frequently colonized by the asthma-associated pathogen *C. difficile* [56]*. *Studies have reported disturbed fecal microbiota profiles in caesarean section delivered infants beginning at 1 day after birth and persisting to 6 months of age [58–60], with one report documenting microbial differences a full 7 years after delivery [61]. In their 2008 meta-analysis, Thavagnanam et al. reported a 20% increase of asthma in children born by caesarean section [62], but there is considerable heterogeneity among recent studies. For example, a UK medical record linkage study documented that caesarean delivery was not associated with hospital admission for asthma beyond age 1 [63], while a Canadian study found an association with asthma at age 9 though it was limited to first-time caesarean section only [64]. New evidence for the birth mode-microbiota-asthma pathway has recently emerged from a study employing mediation analysis to show that the effects of caesarean delivery on asthma development are mediated by *C. difficile *[53]. As this study was limited to just 5 bacterial species, it is likely that other yet-to-be-identified bacteria also contribute to this pathway for the perinatal programming of asthma.

### 5.2. Exclusivity of Breastfeeding

Following birth, exclusive breastfeeding confers “beneficial” gut microbiota to infants, including increased colonization by Bifidobacteria and reduced prevalence and abundance of *C. difficile *compared to formula-fed infants [56, 57, 65]. These benefits have been attributed to the prebiotic properties of human-milk oligosaccharides [66] or the transfer of intestinal bacteria from mother to infant through breast milk [67]. Indeed, new research indicates that breast milk contains a collection of bacteria more diverse than previously thought [68]. Concurrently, new studies around the world continue to find that breastfeeding protects against recurrent wheeze and asthma in later childhood [69–73]; however, these benefits may not apply when the nursing mother is atopic [43, 44]. This phenomenon may be related to microbiota, since the breast milk of allergic mothers has been reported to contain significantly lower amounts of Bifidobacteria compared with nonallergic mothers, and their infants have concurrently lower counts of fecal Bifidobacteria [74]. The DOHaD paradigm would describe this scenario as a dietary “mismatch”, whereby infants of atopic mothers initially receive low amounts of Bifidobacteria via breast milk, followed by exposure to higher levels of dietary bacteria after weaning. Since Bifidobacteria influence early immune development (including IgA production and cytokine responses) [75, 76], infants who are not sufficiently exposed to Bifidobacteria in breast milk may have inappropriate immune responses to microbial exposures later in childhood, leading to atopic disorders including asthma.

### 5.3. Use of Antibiotics

After breast milk and other nutritional supplements, antibiotics are the next most commonly ingested substances by infants. Antibiotics affect colonization of the intestine by suppressing commensal bacteria and causing the emergence of asthma-associated pathogens such as *C. difficile* [22]. Research shows that antibiotic use in the immediate period after birth can severely alter gut microbiota in infants [56, 77], and evidence from long-term studies suggests that these perturbations could last for months, if not years [78, 79]. Indirect exposure is also relevant, since gut microbial diversity is reduced in infants born to mothers who received antibiotics during pregnancy or while breastfeeding [57]. In parallel, new studies continue to find that early-life antibiotic exposure is associated with increased risk for wheeze or asthma later in childhood [80–83]. This association is upheld when antibiotic exposure occurs *in utero* [84–86], during the neonatal period [87], or through breastfeeding [55]; however, two studies have demonstrated that the antibiotic-asthma association is limited to children who are not already genetically predisposed to the disease [82, 88]. Once again, this phenomenon may be related to microbiota, since infants of atopic mothers inherit low levels of commensal bacteria [74] such that antibiotic exposure would be relatively less disruptive than for infants with “normal” gut microbiota. Infants of atopic mothers may also be more frequently colonized by *C. difficile* [53, 89, 90]; therefore, emergence of this asthma-associated pathogen may not rely on antibiotic disturbance in these children. 

Recently, two systematic reviews have emphasized that the association between antibiotic use and subsequent asthma development is subject to confounding by reverse causation (because antibiotic treatment often occurs in response to respiratory symptoms) and confounding by indication (because respiratory tract infections leading to antibiotic use may be the underlying trigger for asthma development) [91, 92]. Despite potential confounding in many of the studies reviewed, the authors acknowledged that a causal relationship between antibiotic exposure and subsequent asthma development remains plausible, since a significant pooled estimate of effect was observed for studies that adequately adjusted for respiratory infections [92]. Finally, if disturbance of gut microbiota is indeed the mechanism for the antibiotic-asthma association, then the timing, dose, and type of antibiotics are likely to be important. Future studies of large, prospective cohorts that address these details and adjust for respiratory infections are needed to definitively confirm the effect of antibiotic exposure on the perinatal programming of asthma.

### 5.4. Use of Probiotics

Along with a growing appreciation for the role of gut microbiota in immune development and health outcomes, there is increasing interest in the therapeutic potential of probiotics (live, nonpathogenic bacteria that confer health benefits when ingested) for asthma and other immune-related disorders [93]. Studies have shown that administration of probiotics to pregnant women, nursing mothers, or newborns can influence the establishment and composition of infant gut microbiota [94–96]. In parallel, probiotics have shown promising immunomodulatory effects in animal studies, where perinatal maternal supplementation [97] and direct supplementation of neonates [98] have been found to attenuate allergic airway responses in offspring. However, despite this evidence, clinical trials in humans have been highly variable. While there is reasonable evidence that probiotics may be useful in the treatment or prevention of allergic rhinitis [99], there have been no conclusive studies for asthma to date [100]. Recent reports indicate that probiotics had no effect on asthma development [101], airway inflammation [102], or asthma-related events [103]. Thus, while they clearly influence infant gut microbiota, it remains to be determined whether probiotics play a role in the perinatal programming of asthma.

### 5.5. Perinatal Stress

Infants constantly encounter new situations; some of these will induce more stress than others. There is intriguing evidence from animal studies that stressful events during infancy have the capacity to modify gut microbiota. Using rhesus monkeys, Bailey and Coe were the first to report that disruption of the mother-infant bond could alter the intestinal microbiota of infants [104]. This effect was transient, lasting several days after maternal separation, but the same authors later showed that moderate maternal stress during pregnancy could disrupt infant gut microbiota for six months or longer [105]. Rodent studies support these findings, showing that frequent maternal separation in the first weeks of life is associated with altered gut microbiota in adolescence [106, 107]. New research suggests that it may be possible to mitigate maternal stress-induced effects with prebiotic supplementation during the neonatal period [108]; however, epigenetic mechanisms might also be involved, since rat pups of mothers that exhibited more frequent grooming and licking were found to have differences in DNA methylation, compared to the offspring of less attentive mothers [109]. Although human intestinal microbiome changes have been noted following emotional stress in adults [110, 111], stress-microbiome pathways have not been explored in infants.

Solid evidence exists for the association of stress and asthma. As shown in several studies conducted by Miller and Chen, stressful life events and a harsh family climate in early life can have long-term effects, resulting in elevated proinflammatory cytokines and glucocorticoid resistance in adolescents [112, 113]. Studies of allergic immune profiles in cord blood indicate that prenatal maternal stress modulates fetal innate and adaptive immune responses [16]. In addition, maternal anxiety during pregnancy [114] or parental stress during infancy [115] have been found to increase the likelihood of asthma at school age. It remains to be seen whether gut microbiota, and/or epigenetic mechanisms, are involved in these associations.

## 6. Summary

In this paper, we have presented evidence for the perinatal programming of asthma via the intestinal microbiome—a relatively new perspective that has evolved alongside modern technologies for the study of microbial communities. While epigenetic mechanisms continue to provide new explanations for the DOHaD theory of asthma development, it is increasingly apparent that the intestinal microbiota plays an independent and potentially interactive role. Commensal gut bacteria are essential to immune system development, and exposures disrupting the infant gut microbiota have been linked to asthma. Well-designed prospective birth cohort studies will be required to fully characterize the longstanding impact of caesarean delivery, breastfeeding, antibiotics, probiotics, and perinatal stress on asthma development and to empirically validate the “microflora programming hypothesis” in this context. 

## Figures and Tables

**Table 1:** Summary of perinatal exposures that may influence the programming of asthma via modification of infant gut microbiota.

Perinatal exposure	Effect on gut microbiota	Effect on asthma development
Caesarean delivery	Prevents exposure to maternal fecal microbes. ↓ Bifidobacteria and Bacteroides [57, 58], ↑ *C. difficile *[53, 56]. Differences may persist for years [58–61].	Increases risk of asthma [62]; recent studies inconsistent [53, 63, 64].

Breastfeeding	Confers beneficial gut microbiota through prebiotic properties [66] or direct transfer of bacteria [67, 68]. ↑ Bifidobacteria, ↓*C. difficile* [56, 57, 65].	Protects against asthma [69–73], except when mother is atopic [43, 44].

Antibiotics	Suppresses commensal bacteria, permits emergence of *C. difficile* [22, 56, 77]. Disturbance may persist for years [78, 79]. Even indirect exposure is harmful [57].	Increases risk of asthma [80–83], except when parents are atopic [82, 88]. Even indirect exposure is harmful [55, 84–87]. Some studies may be confounded [91, 92].

Probiotics	Direct or indirect exposure beneficially influences gut microbiota composition [94–96].	Protects against asthma in animal studies [97, 98]; human trials inconclusive [100–103].

Perinatal stress	Causes transient and long-lasting changes to gut microbiota in animal studies [104–107].	Increases risk of asthma [114, 115].

“Indirect exposure” refers to exposure occurring via the mother, during pregnancy or lactation.

## References

[1] Von Mutius E (2010). 99th Dahlem conference on infection, inflammation and chronic inflammatory disorders: farm lifestyles and the hygiene hypothesis. *Clinical and Experimental Immunology*.

[2] Shreiner A, Huffnagle GB, Noverr MC (2008). The “microflora hypothesis” of allergic disease. *Advances in Experimental Medicine and Biology*.

[3] Wadhwa PD, Buss C, Entringer S, Swanson JM (2009). Developmental origins of health and disease: brief history of the approach and current focus on epigenetic mechanisms. *Seminars in Reproductive Medicine*.

[4] Waterland RA, Michels KB (2007). Epigenetic epidemiology of the developmental origins hypothesis. *Annual Review of Nutrition*.

[5] Barker DJP (2007). The origins of the developmental origins theory. *Journal of Internal Medicine*.

[6] Gluckman PD, Hanson MA, Beedle AS (2007). Early life events and their consequences for later disease: a life history and evolutionary perspective. *American Journal of Human Biology*.

[7] Hanson M, Godfrey KM, Lillycrop KA, Burdge GC, Gluckman PD (2011). Developmental plasticity and developmental origins of non-communicable disease: theoretical considerations and epigenetic mechanisms. *Progress in Biophysics and Molecular Biology*.

[8] Dolinoy DC, Weidman JR, Waterland RA, Jirtle RL (2006). Maternal genistein alters coat color and protects Avy mouse offspring from obesity by modifying the fetal epigenome. *Environmental Health Perspectives*.

[9] Attig L, Gabory A, Junien C (2010). Early nutrition and epigenetic programming: chasing shadows. *Current Opinion in Clinical Nutrition and Metabolic Care*.

[10] Martino D, Prescott S (2011). Epigenetics and prenatal influences on asthma and allergic airways disease. *Chest*.

[11] Drever N, Saade GR, Bytautiene E (2010). Fetal programming: early-life modulations that affect adult outcomes. *Current Allergy and Asthma Reports*.

[12] Kozyrskyj AL, Bahreinian S, Azad MB (2011). Early life exposures: impact on asthma and allergic disease. *Current Opinion in Allergy and Clinical Immunology*.

[13] Hylkema MN, Blacquiere MJ (2009). Intrauterine effects of maternal smoking on sensitization, asthma, and chronic obstructive pulmonary disease. *Proceedings of the American Thoracic Society*.

[14] Noakes PS, Holt PG, Prescott SL (2003). Maternal smoking in pregnancy alters neonatal cytokine responses. *Allergy*.

[15] Chatzi L, Torrent M, Romieu I (2008). Mediterranean diet in pregnancy is protective for wheeze and atopy in childhood. *Thorax*.

[16] Wright RJ, Visness CM, Calatroni A (2010). Prenatal maternal stress and cord blood innate and adaptive cytokine responses in an inner-city cohort. *American Journal of Respiratory and Critical Care Medicine*.

[17] Breton CV, Byun HM, Wenten M, Pan F, Yang A, Gilliland FD (2009). Prenatal tobacco smoke exposure affects global and gene-specific DNA methylation. *American Journal of Respiratory and Critical Care Medicine*.

[18] Pfefferle PI, Pinkenburg O, Renz H (2010). Fetal epigenetic mechanisms and innate immunity in asthma. *Current Allergy and Asthma Reports*.

[19] Martino DJ, Prescott SL (2010). Silent mysteries: epigenetic paradigms could hold the key to conquering the epidemic of allergy and immune disease. *Allergy*.

[20] Breton CV, Byun HM, Wang X, Salam MT, Siegmund K, Gilliland FD (2011). Methylation in the ARG-NOS pathway is associated with exhaled nitric oxide in asthmatic children. *American Journal of Respiratory and Critical Care Medicine*.

[21] Su RC, Becker AB, Kozyrskyj AL, Hayglass KT (2009). Altered epigenetic regulation and increasing severity of bronchial hyperresponsiveness in atopic asthmatic children. *Journal of Allergy and Clinical Immunology*.

[22] Torrazza RM, Neu J (2011). The developing intestinal microbiome and its relationship to health and disease in the neonate. *Journal of Perinatology*.

[23] Vael C, Desager K (2009). The importance of the development of the intestinal microbiota in infancy. *Current Opinion in Pediatrics*.

[24] Kaplan JL, Shi HN, Walker WA (2011). The role of microbes in developmental immunologic programming. *Pediatric Research*.

[25] Marques TM, Wall R, Ross RP, Fitzgerald GF, Ryan CA, Stanton C (2010). Programming infant gut microbiota: influence of dietary and environmental factors. *Current Opinion in Biotechnology*.

[26] Stites DP, Pavia CS (1979). Ontogeny of human T cells. *Pediatrics*.

[27] Pegrum GD (1971). Mixed culture of human foetal and adult cells. *Immunology*.

[28] Jones AC, Miles EA, Warner JO, Colwell BM, Bryant TN, Warner JA (1996). Fetal peripheral blood mononuclear cell proliferative responses to mitogenic and allergenic stimuli during gestation. *Pediatric Allergy and Immunology*.

[29] Prescott SL, Macaubas C, Holt BJ (1998). Transplacental priming of the human immune system to environmental allergens: universal skewing of initial T cell responses toward the Th2 cytokine profile. *Journal of Immunology*.

[30] Holt PG, Upham JW, Sly PD (2005). Contemporaneous maturation of immunologic and respiratory functions during early childhood: implications for development of asthma prevention strategies. *Journal of Allergy and Clinical Immunology*.

[31] Weinberg ED (1984). Pregnancy-associated depression of cell-mediated immunity. *Reviews of Infectious Diseases*.

[32] Jones CA, Finlay-Jones JJ, Hart PH (1997). Type-1 and type-2 cytokines in human late-gestation decidual tissue. *Biology of Reproduction*.

[33] Wegmann TG, Lin H, Guilbert L, Mosmann TR (1993). Bidirectional cytokine interactions in the maternal-fetal relationship: is successful pregnancy a TH2 phenomenon?. *Immunology Today*.

[34] Barrett EG (2008). Maternal influence in the transmission of asthma susceptibility. *Pulmonary Pharmacology and Therapeutics*.

[35] LeBouder E, Rey-Nores JE, Raby AC (2006). Modulation of neonatal microbial recognition: TLR-mediated innate immune responses are specifically and differentially modulated by human milk. *Journal of Immunology*.

[36] Field CJ, Van Aerde JE, Robinson LE, Clandinin MT (2008). Feeding a formula supplemented with long chain polyunsaturated fatty acids modifies the “ex vivo” cytokine responses to food proteins in infants at low risk for allergy. *Pediatric Research*.

[37] Midodzi WK, Rowe BH, Majaesic CM, Saunders LD, Senthilselvan A (2010). Early life factors associated with incidence of physician-diagnosed asthma in preschool children: results from the canadian early childhood development cohort study. *Journal of Asthma*.

[38] Sonnenschein-van der Voort AM, Jaddoe VV, van der Valk RJ Duration and exclusiveness of breastfeeding and childhood asthma-related symptoms.

[39] Gonzalez J, Fernandez M, Garcia FL (2010). Exclusive breastfeeding reduces asthma in a group of children from the Caguas municipality of Puerto Rico. *Boletín de la Asociación Médica de Puerto Rico*.

[40] Mai XM, Becker AB, Sellers EAC, Liem JJ, Kozyrskyj AL (2007). The relationship of breast-feeding, overweight, and asthma in preadolescents. *Journal of Allergy and Clinical Immunology*.

[41] Bjorksten B, Ait-Khaled N, Innes AM, Clayton TO, Robertson C Global analysis of breast feeding and risk of symptoms of asthma, rhinoconjunctivitis and eczema in 6-7 year old children: ISAAC phase three.

[42] Elliott L, Henderson J, Northstone K, Chiu GY, Dunson D, London SJ (2008). Prospective study of breast-feeding in relation to wheeze, atopy, and bronchial hyperresponsiveness in the Avon longitudinal study of parents and children (ALSPAC). *Journal of Allergy and Clinical Immunology*.

[43] Wright AL, Holberg CJ, Taussig LM, Martinez F (2000). Material asthma status alters relation of infant feeding to asthma childhood. *Advances in Experimental Medicine and Biology*.

[44] Pohlabeln H, Mühlenbruch K, Jacobs S, Bohmann H (2010). Frequency of allergic diseases in 2-year-old children in relationship to parental history of allergy and breastfeeding. *Journal of Investigational Allergology and Clinical Immunology*.

[45] Leme AS, Hubeau C, Xiang Y (2006). Role of breast milk in a mouse model of maternal transmission of asthma susceptibility. *Journal of Immunology*.

[46] Licciardi PV, Wong SS, Tang ML, Karagiannis TC (2010). Epigenome targeting by probiotic metabolites. *Gut Pathogens*.

[47] Venema K (2010). Role of gut microbiota in the control of energy and carbohydrate metabolism. *Current Opinion in Clinical Nutrition and Metabolic Care*.

[48] Allan K, Devereux G (2011). Diet and asthma: nutrition implications from prevention to treatment. *Journal of the American Dietetic Association*.

[49] Kalliomäki M, Kirjavainen P, Eerola E, Kero P, Salminen S, Isolauri E (2001). Distinct patterns of neonatal gut microflora in infants in whom atopy was and was not developing. *Journal of Allergy and Clinical Immunology*.

[50] Vebo HC, Sekelja M, Nestestog R (2011). Temporal development of the infant gut microbiota in IgE sensitized and non-sensitized children determined by the GA-map infant array. *Clinical and Vaccine Immunolog*.

[51] Penders J, Thijs C, van den Brandt PA (2007). Gut microbiota composition and development of atopic manifestations in infancy: the KOALA birth cohort study. *Gut*.

[52] Vael C, Vanheirstraeten L, Desager KN, Goossens H (2011). Denaturing gradient gel electrophoresis of neonatal intestinal microbiota in relation to the development of asthma. *BMC Microbiology*.

[53] van Nimwegen FA, Penders J, Stobberingh EE Mode and place of delivery, gastrointestinal microbiota, and their influence on asthma and atopy.

[54] Arumugam M, Raes J, Pelletier E (2011). Enterotypes of the human gut microbiome. *Nature*.

[55] Kummeling I, Stelma FF, Dagnelie PC (2007). Early life exposure to antibiotics and the subsequent development of eczema, wheeze, and allergic sensitization in the first 2 years of life: the KOALA birth cohort study. *Pediatrics*.

[56] Penders J, Thijs C, Vink C (2006). Factors influencing the composition of the intestinal microbiota in early infancy. *Pediatrics*.

[57] Fallani M, Young D, Scott J (2010). Intestinal microbiota of 6-week-old infants across Europe: geographic influence beyond delivery mode, breast-feeding, and antibiotics. *Journal of Pediatric Gastroenterology and Nutrition*.

[58] Gronlund MM, Lehtonen OP, Eerola E, Kero P (1999). Fecal microflora in healthy infants born by different methods of delivery: permanent changes in intestinal flora after cesarean delivery. *Journal of Pediatric Gastroenterology and Nutrition*.

[59] Biasucci G, Rubini M, Riboni S, Morelli L, Bessi E, Retetangos C (2010). Mode of delivery affects the bacterial community in the newborn gut. *Early Human Development*.

[60] Dominguez-Bello MG, Costello EK, Contreras M (2010). Delivery mode shapes the acquisition and structure of the initial microbiota across multiple body habitats in newborns. *Proceedings of the National Academy of Sciences of the United States of America*.

[61] Salminen S, Gibson GR, McCartney AL, Isolauri E (2004). Influence of mode of delivery on gut microbiota composition in seven year old children. *Gut*.

[62] Thavagnanam S, Fleming J, Bromley A, Shields MD, Cardwell CR (2008). A meta-analysis of the association between Caesarean section and childhood asthma. *Clinical and Experimental Allergy*.

[63] Davidson R, Roberts SE, Wotton CJ, Goldacre MJ (2010). Influence of maternal and perinatal factors on subsequent hospitalisation for asthma in children: evidence from the Oxford record linkage study. *BMC Pulmonary Medicine*.

[64] Marra F, Marra CA, Richardson K (2009). Antibiotic use in children is associated with increased risk of asthma. *Pediatrics*.

[65] Roger LC, McCartney AL (2010). Longitudinal investigation of the faecal microbiota of healthy full-term infants using fluorescence in situ hybridization and denaturing gradient gel electrophoresis. *Microbiology*.

[66] Shen Q, Tuohy KM, Gibson GR, Ward RE (2011). In vitro measurement of the impact of human milk oligosaccharides on the faecal microbiota of weaned formula-fed infants compared to a mixture of prebiotic fructooligosaccharides and galactooligosaccharides. *Letters in Applied Microbiology*.

[67] Albesharat R, Ehrmann MA, Korakli M, Yazaji S, Vogel RF (2011). Phenotypic and genotypic analyses of lactic acid bacteria in local fermented food, breast milk and faeces of mothers and their babies. *Systematic and Applied Microbiology*.

[68] Hunt KM, Foster JA, Forney LJ (2011). Characterization of the diversity and temporal stability of bacterial communities in human milk. *PLoS One*.

[69] Garcia-Marcos L, Mallol J, Sole D, Brand PLP (2010). International study of wheezing in infants: risk factors in affluent and non-affluent countries during the first year of life. *Pediatric Allergy and Immunology*.

[70] Kull I, Melen E, Alm J (2010). Breast-feeding in relation to asthma, lung function, and sensitization in young schoolchildren. *Journal of Allergy and Clinical Immunology*.

[71] Demir AU, Celikel S, Karakaya G, Kalyoncu AF (2010). Asthma and allergic diseases in school children from 1992 to 2007 with incidence data. *Journal of Asthma*.

[72] Kusunoki T, Morimoto T, Nishikomori R (2010). Breastfeeding and the prevalence of allergic diseases in schoolchildren: does reverse causation matter?. *Pediatric Allergy and Immunology*.

[73] Just J, Belfar S, Wanin S, Pribil C, Grimfeld A, Duru G (2010). Impact of innate and environmental factors on wheezing persistence during childhood. *Journal of Asthma*.

[74] Gronlund MM, Gueimonde M, Laitinen K (2007). Maternal breast-milk and intestinal bifidobacteria guide the compositional development of the bifidobacterium microbiota in infants at risk of allergic disease. *Clinical and Experimental Allergy*.

[75] Sjogren YM, Tomicic S, Lundberg A (2009). Influence of early gut microbiota on the maturation of childhood mucosal and systemic immune responses. *Clinical and Experimental Allergy*.

[76] Martino DJ, Currie H, Taylor A, Conway P, Prescott SL (2008). Relationship between early intestinal colonization, mucosal immunoglobulin a production and systemic immune development. *Clinical and Experimental Allergy*.

[77] Tanaka S, Kobayashi T, Songjinda P (2009). Influence of antibiotic exposure in the early postnatal period on the development of intestinal microbiota. *FEMS Immunology and Medical Microbiology*.

[78] Dethlefsen L, Huse S, Sogin ML, Relman DA (2008). The pervasive effects of an antibiotic on the human gut microbiota, as revealed by deep 16s rRNA sequencing. *PLoS Biology*.

[79] Jernberg C, Lofmark S, Edlund C, Jansson JK (2007). Long-term ecological impacts of antibiotic administration on the human intestinal microbiota. *ISME Journal*.

[80] Kwon JW, Kim BJ, Song Y (2010). Changes in the prevalence of childhood asthma in Seoul from 1995 to 2008 and its risk factors. *Allergy, Asthma and Immunology Research*.

[81] Mai XM, Kull I, Wickman M, Bergstrom A (2010). Antibiotic use in early life and development of allergic diseases: respiratory infection as the explanation. *Clinical and Experimental Allergy*.

[82] Risnes KR, Belanger K, Murk W, Bracken MB (2011). Antibiotic exposure by 6 months and asthma and allergy at 6 years: findings in a cohort of 1,401 US children. *American Journal of Epidemiology*.

[83] Jedrychowski W, Perera F, Maugeri U (2011). Wheezing and asthmamay be enhanced by broad spectrum antibiotics used in early childhood. Concept and results of a pharmacoepidemiology study. *Journal of Physiology and Pharmacology*.

[84] Jedrychowski W, Galas A, Whyatt R, Perera F (2006). The prenatal use of antibiotics and the development of allergic disease in one year old infants. A preliminary study. *International Journal of Occupational Medicine and Environmental Health*.

[85] Martel MJ, Rey E, Malo JL (2009). Determinants of the incidence of childhood asthma: a two-stage case-control study. *American Journal of Epidemiology*.

[86] McKeever TM, Lewis SA, Smith C, Hubbard R (2002). The importance of prenatal exposures on the development of allergic disease: a birth cohort study using the West Midlands general practice database. *American Journal of Respiratory and Critical Care Medicine*.

[87] Alm B, Erdes L, Mollborg P (2008). Neonatal antibiotic treatment is a risk factor for early wheezing. *Pediatrics*.

[88] Kozyrskyj AL, Ernst P, Becker AB (2007). Increased risk of childhood asthma from antibiotic use in early life. *Chest*.

[89] Adlerberth I, Lindberg E, Aberg N (2006). Reduced enterobacterial and increased staphylococcal colonization of the infantile bowel: an effect of hygienic lifestyle?. *Pediatric Research*.

[90] Johansson MA, Sjogren YM, Persson JO, Nilsson C, Sverremark-Ekstrom E (2011). Early colonization with a group of Lactobacilli decreases the risk for allergy at five years of age despite allergic heredity. *PLoS One*.

[91] Penders J, Kummeling I, Thijs C (2011). Infant antibiotic use and wheeze and asthma risk: a systematic review and meta-analysis. *European Respiratory Journal*.

[92] Murk W, Risnes KR, Bracken MB (2011). Prenatal or early-life exposure to antibiotics and risk of childhood asthma: a systematic review. *Pediatrics*.

[93] Forsythe P (2011). Probiotics and lung diseases. *Chest*.

[94] Kukkonen K, Savilahti E, Haahtela T (2007). Probiotics and prebiotic galacto-oligosaccharides in the prevention of allergic diseases: a randomized, double-blind, placebo-controlled trial. *Journal of Allergy and Clinical Immunology*.

[95] Gueimonde M, Sakata S, Kalliomaki M, Isolauri E, Benno Y, Salminen S (2006). Effect of maternal consumption of lactobacillus GG on transfer and establishment of fecal bifidobacterial microbiota in neonates. *Journal of Pediatric Gastroenterology and Nutrition*.

[96] Schultz M, Gottl C, Young RJ, Iwen P, Vanderhoof JA (2004). Administration of oral probiotic bacteria to pregnant women causes temporary infantile colonization. *Journal of Pediatric Gastroenterology and Nutrition*.

[97] Blumer N, Sel S, Virna S (2007). Perinatal maternal application of Lactobacillus rhamnosus GG suppresses allergic airway inflammation in mouse offspring. *Clinical and Experimental Allergy*.

[98] Feleszko W, Jaworska J, Rha RD (2007). Probiotic-induced suppression of allergic sensitization and airway inflammation is associated with an increase of T regulatory-dependent mechanisms in a murine model of asthma. *Clinical and Experimental Allergy*.

[99] Singh M, Das DR (2010). Probiotics for allergic respiratory diseases—putting it into perspective. *Pediatric Allergy and Immunology*.

[100] Sanz Y Gut microbiota and probiotics in maternal and infant health.

[101] Dotterud CK, Storro O, Johnsen R, Oien T (2010). Probiotics in pregnant women to prevent allergic disease: a randomized, double-blind trial. *British Journal of Dermatology*.

[102] Kukkonen AK, Kuitunen M, Savilahti E, Pelkonen A, Malmberg P, Makela M (2011). Airway inflammation in probiotic-treated children at 5 years. *Pediatric Allergy and Immunology*.

[103] Rose MA, Stieglitz F, Koksal A, Schubert R, Schulze J, Zielen S (2010). Efficacy of probiotic lactobacillus GG on allergic sensitization and asthma in infants at risk. *Clinical and Experimental Allergy*.

[104] Bailey MT, Coe CL (1999). Maternal separaseparation disrupts the integrity of the intestinal microflora in infant rhesus monkeys. *Developmental Psychobiology*.

[105] Bailey MT, Lubach GR, Coe CL (2004). Prenatal stress alters bacterial colonization of the gut in infant monkeys. *Journal of Pediatric Gastroenterology and Nutrition*.

[106] Garcia-Rodenas CL, Bergonzelli GE, Nutten S (2006). Nutritional approach to restore impaired intestinal barrier function and growth after neonatal stress in rats. *Journal of Pediatric Gastroenterology and Nutrition*.

[107] O’Mahony SM, Marchesi JR, Scully P (2009). Early life stress alters behavior, immunity, and microbiota in rats: implications for irritable bowel syndrome and psychiatric illnesses. *Biological Psychiatry*.

[108] Gareau MG, Jury J, MacQueen G, Sherman PM, Perdue MH (2007). Probiotic treatment of rat pups normalises corticosterone release and ameliorates colonic dysfunction induced by maternal separation. *Gut*.

[109] Weaver ICG, Cervoni N, Champagne FA (2004). Epigenetic programming by maternal behavior. *Nature Neuroscience*.

[110] Lutgendorff F, Akkermans LMA, Soderholm JD (2008). The role of microbiota and probiotics in stress-induced gastrointestinal damage. *Current Molecular Medicine*.

[111] Holdeman LV, Good IJ, Moore WEC (1976). Human fecal flora: variation in bacterial composition within individuals and a possible effect of emotional stress. *Applied and Environmental Microbiology*.

[112] Chen E, Hanson MD, Paterson LQ, Griffin MJ, Walker HA, Miller GE (2006). Socioeconomic status and inflammatory processes in childhood asthma: the role of psychological stress. *Journal of Allergy and Clinical Immunology*.

[113] Miller GE, Chen E (2010). Harsh family climate in early life presages the emergence of a proinflammatory phenotype in adolescence. *Psychological Science*.

[114] Cookson H, Granell R, Joinson C, Ben-Shlomo Y, Henderson AJ (2009). Mothers’ anxiety during pregnancy is associated with asthma in their children. *Journal of Allergy and Clinical Immunology*.

[115] Lange NE, Bunyavanich S, Silberg JL, Canino G, Rosner BA, Celedon JC (2011). Parental psychosocial stress and asthma morbidity in Puerto Rican twins. *Journal of Allergy and Clinical Immunology*.

